# Therapeutic exploitation of IPSE, a urogenital parasite-derived host modulatory protein, for chemotherapy-induced hemorrhagic cystitis

**DOI:** 10.1096/fj.201701415R

**Published:** 2018-04-03

**Authors:** Evaristus C. Mbanefo, Loc Le, Luke F. Pennington, Justin I. Odegaard, Theodore S. Jardetzky, Abdulaziz Alouffi, Franco H. Falcone, Michael H. Hsieh

**Affiliations:** * Bladder Immunology Group, Biomedical Research Institute, Rockville, Maryland, USA;; † Division of Urology, Children’s National Medical Center, Washington, District of Columbia, USA;; ‡ Department of Structural Biology, Stanford University School of Medicine, Stanford, California, USA;; § OneOme, Redwood City, California, USA;; ¶ Life Science and Environment Sector, King Abdulaziz City for Science and Technology, Riyadh, Saudi Arabia;; ‖ Division of Molecular Therapeutics and Formulation, School of Pharmacy, University of Nottingham, Nottingham, United Kingdom;; # Department of Urology, The George Washington University, Washington, District of Columbia, USA

**Keywords:** infiltrin, anti-inflammatory, ifosfamide, urothelial, *Schistosoma*

## Abstract

Chemotherapy-induced hemorrhagic cystitis (CHC) can be difficult to manage. Prior work suggests that IL-4 alleviates ifosfamide-induced hemorrhagic cystitis (IHC), but systemically administered IL-4 causes significant side effects. We hypothesized that the *Schistosoma hematobium* homolog of IL-4-inducing principle from *Schistosoma mansoni* eggs (H-IPSE), would reduce IHC and associated bladder pathology. IPSE binds IgE on basophils and mast cells, triggering IL-4 secretion by these cells. IPSE is also an “infiltrin,” translocating into the host nucleus to modulate gene transcription. Mice were administered IL-4, H-IPSE protein or its nuclear localization sequence (NLS) mutant, with or without neutralizing anti-IL-4 antibody, or 2-mercaptoethane sulfonate sodium (MESNA; a drug used to prevent IHC), followed by ifosfamide. Bladder tissue damage and hemoglobin content were measured. Spontaneous and evoked pain, urinary frequency, and bladdergene expression analysis were assessed. Pain behaviors were interpreted in a blinded fashion. One dose of H-IPSE was superior to MESNA and IL-4 in suppressing bladder hemorrhage in an IL-4-dependent fashion and comparable with MESNA in dampening ifosfamide-triggered pain behaviors in an NLS-dependent manner. H-IPSE also accelerated urothelial repair following IHC. Our work represents the first therapeutic exploitation of a uropathogen-derived host modulatory molecule in a clinically relevant bladder disease model and indicates that IPSE may be an alternative to MESNA for mitigating CHC.—Mbanefo, E. C., Le, L., Pennington, L. F., Odegaard, J. I., Jardetzky, T. S., Alouffi, A., Falcone, F. H., Hsieh, M. H. Therapeutic exploitation of IPSE, a urogenital parasite-derived host modulatory protein, for chemotherapy-induced hemorrhagic cystitis.

Hemorrhagic cystitis can result as a pathogenic feature of infection, radiation therapy, and chemotherapy. The most common inciting agents for the latter are cyclophosphamide and ifosfamide, nitrogen mustard alkylating agents used to treat many cancers (1). Liver metabolism of cyclophosphamide and ifosfamide generates acrolein, which concentrates in the urine, activating cascades that can lead to hemorrhagic cystitis (2). Indeed, toxicity in the form of hemorrhagic cystitis can be a dose-limiting factor for these drugs (3). Up to 40% of patients receiving cyclophosphamide and ifosfamide experience some degree of hemorrhagic cystitis (4), which can result in major chronic sequelae, such as hematuria, urinary frequency, stranguria, small volume voids, bladder spasms, reduced bladder capacity, and increased risk of development of secondary bladder cancer. 2-Mercaptoethane sulfonate sodium (MESNA) is given to prevent cyclophosphamide- and ifosfamide-associated hemorrhagic cystitis. This drug works by scavenging acrolein, a highly reactive nucleophile metabolite of ifosfamide, which concentrates in bladder urine and causes tissue damage. However, MESNA fails to treat established hemorrhagic cystitis (5, 6). Moreover, its use can result in systemic hypersensitivity reactions, including arrhythmias (7). In studies of MESNA- and ifosfamide-exposed bladders, cystoscopy and histology show that two-thirds and 100% of patients, respectively, still have evidence of significant bladder injury despite MESNA prophylaxis (8). Options for MESNA-refractory, cyclophosphamide-induced hemorrhagic cystitis and ifosfamide-induced hemorrhagic cystitis (IHC) are limited and suboptimal (9).

Cytokines, particularly IL-4, appear to play an important role in chemotherapy-induced hemorrhagic cystitis (CHC) and thus, are promising therapeutic strategies. Administration of recombinant IL-4 alleviates IHC *via* attenuation of bladder TNF-α, IL-1β, iNOS, and cyclooxygenase 2 (COX-2) (10), which are proinflammatory mediators previously associated with hemorrhagic cystitis (2, 11–16). Interestingly, exposure of mice to ifosfamide induces endogenous IL-4 production, suggesting that there are intrinsic, IL-4-related homeostatic responses to this chemotherapeutic agent (10). Accordingly, both antibody- and transgenic-based abrogation of IL-4 activity in mice results in more severe IHC (10). However, systemic administration of IL-4 is unlikely to be an acceptable prophylactic or therapeutic approach for cyclophosphamide-induced hemorrhagic cystitis and IHC. For instance, clinical trials of systemic administration of IL-4 for cancer therapy have resulted in untoward side effects (17–28). Nevertheless, evidence suggests that IL-4-associated anti-inflammatory pathways and targets thereof are promising therapeutic candidates for hemorrhagic cystitis.

The *Schistosoma hematobium* homolog of IL-4-inducing principle from *Schistosoma mansoni* eggs (H-IPSE) is a molecule that may modulate *S. hematobium*-associated hemorrhagic cystitis and broader host bladder biology. Like ifosfamide, *S. hematobium* infection is associated with a form of hemorrhagic cystitis, thought to be chiefly caused by chronic inflammation induced by eggs deposited in the bladder wall and by passage of eggs through the bladder and into the urinary stream. IPSE is the most abundant parasite egg-secreted protein, and no homolog exists outside of the genus *Schistosoma* (29). Chronic *S. hematobium* infection, but not *S. mansoni* infection, is associated with hematuria. However, only a minority of infected individuals shows day-to-day hematuria or other urinary symptoms (30). Whereas other factors, including intensity of infection, are associated with the degree of hematuria, it is possible that a parasite molecule-induced modulatory effect may play a role in this pathogenesis. Thus, we reasoned that H-IPSE may have evolved as species-specific molecules responsible for carefully calibrating host hemorrhagic cystitis, which in controlled degrees, may be necessary for egg passage but is detrimental to host (and thus, parasite) when excessive morbidity results (31). *S. mansoni* IPSE (M-IPSE) binds Igs, in particular, IgE, on basophils and mast cells, which triggers IL-4 secretion (32–34). M-IPSE also sequesters chemokines (35) and is an infiltrin that relies on a nuclear localization sequence (NLS) to enter host cell nuclei to alter gene transcription (36, 37). We hypothesized that similar IL-4-inducing and NLS-dependent functions exist in H-IPSE (38), and that these properties may alleviate IHC. Indeed, we found that a single intravenous dose of H-IPSE was superior to MESNA and IL-4 in suppressing ifosfamide-induced bladder hemorrhage in mice and similar in efficacy to these agents in its ability to dampen ifosfamide-induced pain and abnormal voiding patterns. These features were variably IL-4 and NLS dependent.

## MATERIALS AND METHODS

### Study approval

All animal work has been conducted according to relevant U.S. and international guidelines. Specifically, animal experimental protocols were reviewed and approved by the Institutional Animal Care and Use Committee of the Biomedical Research Institute (Rockville, MD, USA). Our Institutional Animal Care and Use Committee guidelines comply with the U.S. Public Health Service Policy on Humane Care and Use of Laboratory Animals.

### Animals, reagents, and drugs

Female 6- to 8-wk-old C57BL/6 mice (Charles River Laboratories, Wilmington, MA, USA) and IL-4/green fluorescent protein (GFP)-enhanced transcript (4get) mice (39) (The Jackson Laboratories, Bar Harbor, ME, USA) were housed under 12 h light-dark cycles in temperature-controlled holding rooms with unlimited access to dry mouse chow and water. Our group has cloned and characterized different variants of H-IPSE (38). Ifosfamide and MESNA (both >98% purity) were purchased from Sigma-Aldrich (St. Louis, MO, USA). Recombinant murine IL-4 was obtained from Peprotech Laboratories (Rocky Hill, NJ, USA). Neutralizing anti-IL-4 mAb (11B11 clone) was purchased from BioXcell (West Lebanon, NH, USA).

### Recombinant protein preparation

The method used for H-IPSE identification, generation of its NLS mutant, cloning, and expression was previously described by our group (38). Material for *in vitro* and *in vivo* experiments underwent the following expression and purification protocol. One milligram of plasmid DNA was purified using a GenElute HP endotoxin-free plasmid Maxiprep kit (Sigma-Aldrich) and was incubated with 3 mg linear 25 kDa polyethylenimine (PolySciences, Warrington, PA, USA) at 1 mg/ml, diluted in 10 ml sterile PBS, pH 7.4, for each 1 L transfection. Secreted recombinant protein was expressed in human embryonic kidney 293-6E cells (40) for 5 d in suspension culture using FreeStyle 293 medium (Thermo Fisher Scientific, Waltham, MA, USA) and purified over 10 ml Ni-NTA resin (Qiagen, Germantown, MD, USA), equilibrated with 10 mM imidazole PBS, pH 7.4, washed with 5 resin bed volumes of 25 mM imidazole PBS, pH 7.4, and eluted with 300 mM imidazole PBS, pH 7.4. The eluted protein was concentrated with a 10 kDa cutoff 15 ml Amicon Ultra Centrifugal Filter Unit (EMD Millipore, Billerica, MA, USA) to a concentration of no >0.5 mg/ml and purified on a Hiload 16/600 Superdex 200 Column (GE Healthcare, Waukesha, WI, USA). Before purification, FPLC machines and Hiload columns were cleaned with 0.5 M NaOH for 1.5 column volumes at a flow rate of 1 ml/min (3 h) and then washed with 1.5 column volumes of PBS, pH 7.4. All buffers were made with MilliQ ddH_2_O to reduce pyrogen contamination, and mutant protein controls were purified on the same day with identical buffers. All protein samples were further filter sterilized by passage through a 0.22-μm syringe filter.

### Hemorrhagic cystitis model

The IHC model used in this study was adapted from methods previously described by Macedo *et al.* (10). Mice were: *1*) intravenously injected with 25 μg H06 H-IPSE or the NLS mutant of H-IPSE, 24 h before intraperitoneal ifosfamide injection (400 mg/kg); *2*) injected with H-IPSE, followed 23.5 h later by intraperitoneal injection of 100 μg neutralizing anti-IL-4 antibody (11B11), given 30 min before ifosfamide injection; *3*) intraperitoneally administered 10 ng recombinant IL-4, 1 h before ifosfamide injection; *4*) given MESNA [80 mg/kg, 20% of ifosfamide dose (10)] just before ifosfamide injection and 2 more doses, 4 h apart; *5*) intraperitoneally administered saline alone; or *6*) injected with saline, followed by ifosfamide (400 mg/kg), 24 h later. Mice were monitored for 12-h postifosfamide injection, before they were euthanized for downstream experiments.

### Gross evaluation of bladders for edema and hemorrhage

Mice were euthanized 12 h postifosfamide injection and their bladders immediately excised, weighed to measure bladder wet weight, and used for downstream experiments. Bladders were also evaluated macroscopically for signs of edema and hemorrhage on a scale of 0 – 3+, following Gray’s scoring criteria (41) and as previously detailed (10). For hemorrhage, normal was scored as 0, dilation of blood vessels was scored as 1+, mucosal hematomas were scored as 2+, and intravesical clots were scored as 3+.

### Bladder histology

Bladders were fixed in 10% neutral-buffered formalin and later dehydrated and embedded in paraffin. Paraffin-embedded bladders were cut into 5-μm sections and then processed for hematoxylin and eosin staining. The stained sections were evaluated microscopically (in a blinded fashion by J.I.O.) for the presence of urothelial denudation, lamina propria edema, hemorrhage, and cellular infiltration.

### Measurement of bladder hemoglobin concentration using Drabkin’s reagent

Drabkin’s assay is a cyanomethemoglobin-based method to assess the hemoglobin content of tissue (bladder) as an objective measure of hemorrhage. The assay was performed according to the manufacturer’s instructions (Sigma-Aldrich) and adapted from Macedo *et al.* (10). In brief, we homogenized whole bladders in Drabkin’s reagent (100 mg tissue/ml reagent), and the homogenates were centrifuged at 10,000 *g* for 10 min. The supernatants were centrifuged a second time at 10,000 *g* for 10 min to ensure complete clearance of debris. Absorbance was then measured at 540 nm on a plate reader. A standard curve for the estimation of hemoglobin content was generated using known amounts of hemoglobin (Cayman Chemical, Ann Arbor, MI, USA).

### Spontaneous pain evaluation

Spontaneous pain behaviors were scored using previously published methods (42–45), as detailed by Leventhal and Strassle (46) and as adapted in our laboratory. In brief, mice were placed individually in fresh cages with new bedding. After acclimatizing for 30 min, mice were evaluated for spontaneous pain characteristics. The spontaneous pain characteristics were scored (in a blinded fashion) by observing the mouse for 1 min for any of these characteristics: normal = 0; piloerection (ruffled fur) = 1; labored breathing = 2; ptosis (drooping of upper eyelid) = 3; Licking of abdomen (not grooming) = 4; rounded back = 5. The sum of any observed behaviors/appearance for each mouse was used as the overall spontaneous pain score.

### Evoked pain evaluation (von Frey monofilament assessment of tactile allodynia)

Evoked pain was estimated in a blinded fashion using von Frey’s monofilaments with the following range of bending forces: 0.008, 0.04, 0.16, 0.4, 0.6, 1.0, 1.4, and 2 g. We adopted the up-down method by Chaplan *et al.* (47), as detailed by Leventhal and Strassle (46) and adapted in our laboratory. Mice were placed on wire mesh in an apparatus constructed using an inverted mouse cage and left to acclimatize for 30 min. From underneath the cage through the wire mesh, the tip of a 0.4 g von Frey monofilament was applied to the hind paw until the filament bent and then held for up to 5 s. Responses include rapid withdrawal, jumping, or licking of the stimulated spot. If no response was observed, the next-higher force fiber was applied until a withdrawal response was observed or until 2-g cutoff was reached. If a withdrawal occurred at the initial stimulation using the 0.4 g force fiber, then the next-lighter force was applied until a bending force of 0.008 g (minimum cutoff) was reached. Subsequently, whenever a response was observed, we continued to a lighter fiber, and if no response was observed, we continued to a stronger fiber, until at least 6 unique withdrawal evaluations were obtained. The 50% withdrawal threshold was calculated using the equation [10^(^*^Xf^* ^+^ *^kδ^*^)^/10,000], where the *k* value of the response pattern was determined according to Dixon (48) and as described by Leventhal and Strassle (46); *Xf* represents the log units value for the last von Frey filament applied. For the set of monofilaments used in this study, δ—standing for the mean difference between stimuli—was 0.285.

### Void spot on a paper assay

The voided spot on paper assay for measuring voiding frequency and voiding patterns was performed, as previously described (49, 50) and according to our own published experience (51, 52). In short, a piece of Whatman No. 1 filter paper was cut to cover the entire bottom surface of a mouse cage. Wire mesh was used to prevent mouse access to the filter paper. Individual mice were left in covered cages with only mouse chow provided. After 4 h, the filter papers were recovered and the urine spots detected by UV transillumination. A standard curve for estimation of urine volume was generated by preparation of filter paper with controlled drops of known, increasing volumes of mouse urine.

### Gene expression analysis by real-time PCR

Bladders were aseptically excised and immediately placed in RNAzol solution in a tube containing zirconium beads for subsequent homogenization of bladder tissues. Total RNA was purified using the RNAzol method, according to the manufacturer’s instructions. With the use of the same concentration of total RNA for all samples (500 ng), cDNA was synthesized using the iScript cDNA Synthesis Kit (Bio-Rad, Hercules, CA, USA). Real-time PCR was performed using the iTaq universal SYBR Green Supermix, following the manufacturer’s protocols. The primers used for real-time PCR are as follows: *uroplakin 1a* (*Up1a*; 5′-TACACCCACCGCGACTATATG-3′ and 5′-CCACAACACTCTTGCTCAATCAT-3′), *Up2* (5′-TGCCCCTGATCCTGATTCTG-3′ and 5′-CAAGGCAATTAACAGGCTTTCTG-3′), *claudin 8* (*Cldn8*; 5′-AGAGCCGCATCTTGCTGAC-3′ and 5′-TCTGATGATGGAATTGGCAACC-3′), *Il-1β* (5′-GAAATGCCACCTTTTGACAGTG-3′ and 5′-TGGATGCTCTCATCAGGACAG-3′), *Tnf-α* (5′-GTGGAACTGGCAGAAGAG-3′ and 5′-CCATAGAACTGATGAGAGG-3′), *iNOS* (5′-ACATCGACCCGTCCACAGTAT-3′ and 5′-CAGAGGGGTAGGCTTGTCTC-3′), *Il-8* (5′-CACCTCAAGAACATCCAGAGCT-3′ and 5′-CAAGCAGAACTGAACTACCATCG-3′), *Ccl3* (5′-CAGCCAGGTGTCATTTTCCT-3′ and 5′-CTGGCTCCAAGACTCTCAGG-3′), *Cox-2* (5′-CAAGGGAGTCTGGAACATTG-3′ and 5′-ACCCAGGTCCTCGCTTATGA-3′), and *glyceraldehyde 3-phosphate dehydrogenase* (5′-AGGTCGGTGTGAACGGATTTG-3′ and 5′-TGTAGACCATGTAGTTGAGGTCA-3′). Relative gene expression was then analyzed using the comparative threshold (*C_t_*) method with fold expression using the formula 2^(−∆∆^*^Ct^*^),^ with glyceraldehyde 3-phosphate dehydrogenase as the internal reference and the saline-only group as the baseline negative control.

### Flow cytometry

Flow cytometry was performed on peripheral blood leukocytes and cells from dissociated bladder tissues from 4get mice to evaluate IL-4 expression, 24 h after intravenous injection of H-IPSE or PBS. In brief, whole blood was collected from 4get mice, followed by red blood cell (RBC) lysis for 10 min using RBC lysis buffer (150 mM NH_4_Cl, 10 mM KHCO_3_, 0.1 mM Na_2_ EDTA, pH 7.2–7.4). Freshly excised bladders were dissociated in tissue-dissociated solution following our previously described method (52), before RBC lysis. The cells were filtered through a 40 μm mesh, and resulting single-cell suspension was counted, and then 10^6^ cells were washed twice using flow cytometry wash buffer (1 time PBS containing 0.02% sodium azide, 1% bovine serum albumin) and blocked using 5 μl (0.5 μg/μl) mouse Fc-blocking antibody (anti-CD16/CD32 mAb) for 30 min at 4°C. The cells were then stained with combinations of anti-CD3-*PE-Cy7*, anti-CD49b-*eFluor*, and anti-c-kit-*allophycocyanin* mAb for basophil and mast cell estimation and anti-CD45-*allophycocyanin* and anti-Ly6G-Pacific Blue mAb for neutrophil detection and then incubated on ice in the dark for 1 h. The stained cells were washed twice, resuspended in 1 ml flow cytometry wash buffer, and acquired on a BD FACSCanto II machine (BD Biosciences San Jose, CA, USA). Flow cytometric analyses were performed using the FlowJo fluorescence-activated cell sorting analysis platform, version 10.1 (FlowJo, Ashland, OR, USA). IL-4 expression was assessed using the FITC channel to detect IL-4-linked GFP expression by 4get mice.

### Statistics

Data analysis was performed on GraphPad Prism (GraphPad Software, La Jolla, CA, USA), v.6.00. For comparison within groups, 1-way ANOVA was performed, and if significant, *post hoc* Student’s *t* tests were then used to perform pairwise comparisons after confirming a normal distribution of the data. All plotted data are individual data points, with error bars representing means ± sd. Statistical significance was designated as *P* < 0.05.

## RESULTS

### H-IPSE (variant H06) induces basophils to release IL-4

**Figure 1:**
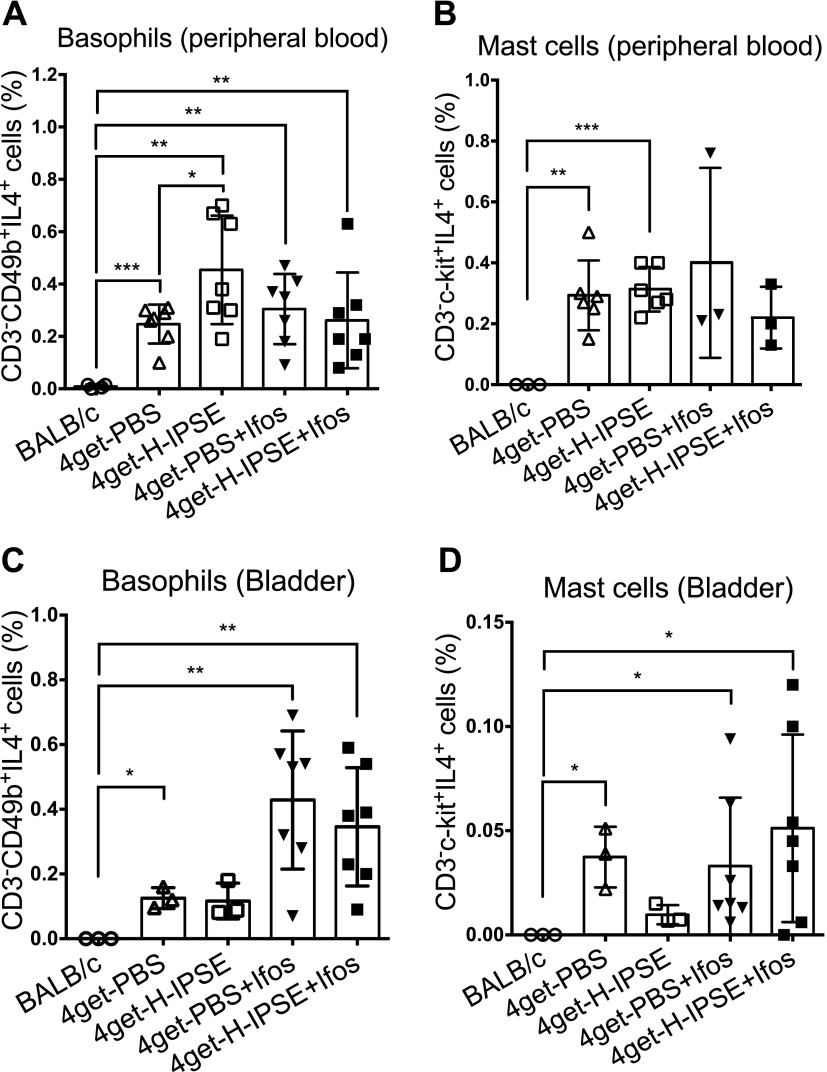
Intravenous injection of H-IPSE induces IL-4 expression by basophils. *A*) A higher proportion of basophils from the peripheral blood of H-IPSE-injected mice were IL-4^+^ compared with PBS-injected mice. After ifosfamide (Ifos) injection, this cell population was similar in both H-IPSE and PBS groups. *B*) There was no significant difference in IL-4^+^ peripheral blood mast cells between H-IPSE and PBS groups before and after ifosfamide injection. *C*) IL-4-expressing basophils were present at higher numbers in the bladders of both H-IPSE and PBS groups after ifosfamide injection. *D*) There was no difference in IL-4-positive bladder mast cells, with or without ifosfamide, between the H-IPSE and PBS groups. Consistently, BALB/c mice did not express GFP (IL-4), as expected. Plotted data are pooled from 2 repeat experiments (*n* = 3–7). Horizontal bars represent means. *P < 0.05, **P < 0.01, ***P < 0.001.

Others have previously reported that M-IPSE binds to IgE, present on FcRs on the surface of basophils and mast cells, thereby inducing IL-4 secretion (53). We used IL-4 transcription-linked GFP expression in 4get mice (39) to confirm that a single intravenous injection of the H06 variant of H-IPSE induces IL-4 transcription in basophils. Supplemental Fig. 1 shows a representative flow cytometric analysis gating strategy for CD3^−^IL-4^+^CD49b^+^ cells, including basophils (Supplemental Fig. 1). CD3^−^IL-4^+^c-kit^+^ expression was used to identify mast cells. BALB/c-negative controls showed no GFP-linked IL-4 expression. IL-4-linked GFP expression was consistently detectable in all 4get mice groups assessed (**Fig. 1**). There was a statistically significant increase (*P* = 0.0394) in IL-4-positive basophils in peripheral blood (Fig. 1*A*) but not mast cells (Fig. 1*B*) from 4get mice intravenously injected with H06 H-IPSE compared with 4get mice receiving PBS. This difference was not observed in cells collected from the bladder (Fig. 1*C, D*). After ifosfamide challenge, the proportion of IL-4-expressing cells in the bladder was increased in both H-IPSE and PBS groups compared with ifosfamide-naive mice. However, there was no difference in the proportion of IL-4-positive basophils and mast cells between the H-IPSE- and PBS-exposed groups after ifosfamide injection.

### H-IPSE reduces ifosfamide-induced bladder hemorrhage and hemoglobin content, but not bladder wet weight and edema, in an IL-4- and NLS-dependent manner

**Figure 2:**
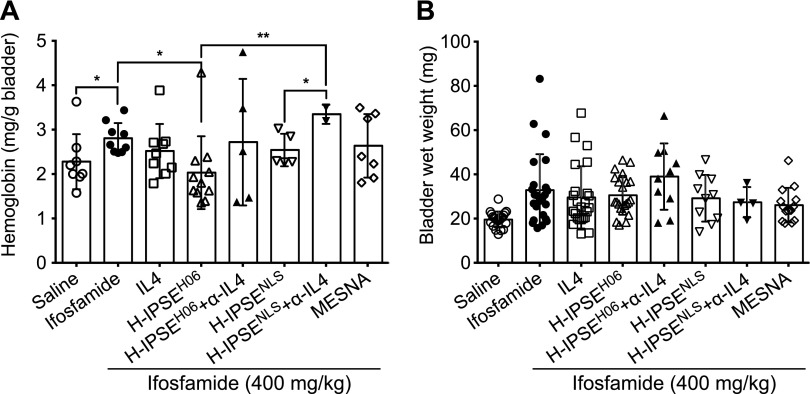
H-IPSE reduces bladder hemoglobin levels, but not bladder wet weight, in ifosfamide-treated mice. *A*) Mice were administered recombinant IL-4, H-IPSE, and the NLS mutant of H-IPSE, with or without neutralizing anti-IL-4 antibody (α-IL4), MESNA, or saline, before ifosfamide challenge. Animals were then euthanized and their bladders excised, weighed to measure bladder wet weight, and then subjected to the Drabkin’s test to measure tissue hemoglobin levels. The H-IPSE-treated group showed significantly reduced bladder hemoglobin levels compared with the ifosfamide-only group, and prior administration of neutralizing anti-IL-4 antibody reversed the effect of H-IPSE, albeit without achieving statistical significance. The NLS mutant-treated group, as well as MESNA-treated mice, did not exhibit a significant decrease in bladder hemoglobin following ifosfamide exposure. Plotted data are pooled from 3 repeat experiments (*n* = 3–4 each). The bars represent means and sd. *B*) There were no significant differences in bladder wet weights across treatment groups. Horizontal bars represent means. *P < 0.05, **P < 0.01.

Having demonstrated that H06 H-IPSE shares several features with M-IPSE, including activating IgE-bearing basophils to produce IL-4 and localizing in host cell nuclei (38), we next sought to determine any *in vivo* effects of H06 H-IPSE in the ifosfamide-induced mouse model of hemorrhagic cystitis. Mice were given saline alone or ifosfamide in combination with the following: 1) an intravenous injection of H06 H-IPSE (wild-type) or an H-IPSE mutant lacking the NLS; 2) H06 H-IPSE or its NLS mutant, followed by neutralizing anti-IL-4 antibody; 3) recombinant IL-4; 4) MESNA; or 5) saline. Compared with mice administered only saline before ifosfamide, mice given H06 H-IPSE showed a significant reduction (42.3%, *P* = 0.0008) in macroscopically apparent bladder hemorrhage. This H-IPSE-mediated reduction in bladder hemorrhage was comparable with the MESNA (42.9%, *P* = 0.0096) and IL-4 (35.3%, *P* = 0.0005) treatment groups (**Table 1**). The observed H06 H-IPSE-mediated reduction in bladder hemorrhage was reversed when neutralizing anti-IL-4 antibody was administered before ifosfamide injection and reduced when the NLS mutant of H-IPSE was given (albeit not statistically significant). Likewise, bladder hemoglobin content was significantly reduced in the H-IPSE-injected group compared with the ifosfamide-only group (*P* = 0.0133; **Fig. 2*A***). Indeed, H-IPSE decreased ifosfamide-induced bladder hemoglobin content better than MESNA and IL-4. This effect of H-IPSE was reversed when neutralizing anti-IL-4 antibody was administered after H-IPSE injection, but the difference was not statistically significant (Fig. 2*A*). Furthermore, relative to the H-IPSE-injected group and although not achieving statistical significance, bladder hemoglobin content was higher in mice given the NLS mutant of H-IPSE. When neutralizing anti-IL-4 antibody was administered to the NLS mutant H-IPSE group, the hemoglobin content was further significantly increased (*P* = 0.002; Fig. 2*A*). Although we noted a decrease in bladder-infiltrating neutrophils in the H06 H-IPSE-treated group compared with the PBS-treated group after ifosfamide injection (Supplemental Fig. 2), all mice receiving ifosfamide, with or without treatments, showed increased bladder wet weight and edema compared with mice not given ifosfamide (Fig. 2*B* and Table 1). Furthermore, compared with the group receiving only saline before ifosfamide, a significant reduction in bladder edema was only observed in mice given MESNA in combination with ifosfamide (*P* = 0.0409).

**TABLE 1:** H-IPSE reduces bladder hemorrhage in ifosfamide-treated mice

	Hemorrhage			Edema		
Experimental group	Mean ± sem	% Reduction	*P*	Mean ± sem	% Reduction	*P*
Saline	0.143 ± 0.078	93.3	<0.0001	0.048 ± 0.048	96.9	<0.0001
Ifosfamide	2.125 ± 0.151	ref	ref	1.542 ± 0.241	Ref	ref
IL-4 + ifosfamide	1.375 ± 0.132	35.3	0.0005	1.167 ± 0.253	24.3	ns
H-IPSE^H06^ + ifosfamide	1.227 ± 0.197	42.3	0.0008	1.273 ± 0.220	17.4	ns
H-IPSE^H06^ + anti-IL-4 antibody + ifosfamide	2.0 ± 0.258	5.9	ns	2.0 ± 0.394	−29.7	ns
H-IPSE^NLS^ + ifosfamide	1.700 ± 0.213	20	ns	1.200 ± 0.359	22.2	ns
H-IPSE^NLS^ + anti-IL-4 antibody + ifosfamide	2.0 ± 0.577	5.9	ns	1.0 ± 0.408	35.1	ns
MESNA + ifosfamide	1.214 ± 0.281	42.9	0.0096	0.786 ± 0.261	49.0	0.0409

Ns, not significant; ref, reference group.

### H-IPSE reduces IL-4-dependent, histologically evident pathologic changes in the ifosfamide-exposed bladders

**Figure 3:**
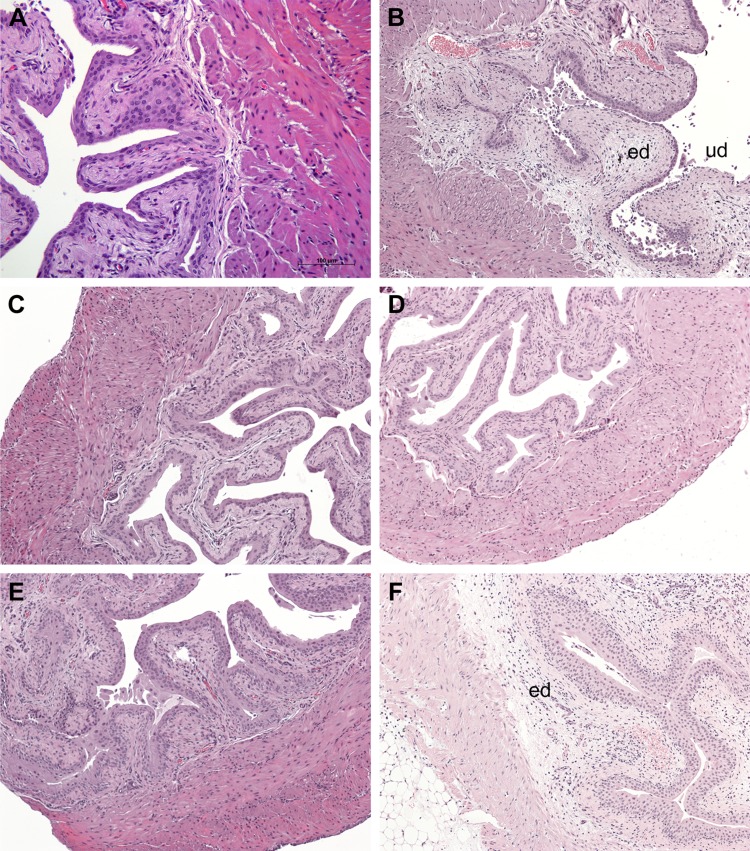
H-IPSE mitigates ifosfamide-induced histopathological changes in the bladder. Histologic assessment of bladders from mice receiving the following: saline only (*A*), ifosfamide only (*B*), MESNA and ifosfamide (*C*), recombinant IL-4 and ifosfamide (*D*), H-IPSE and ifosfamide (*E*), and H-IPSE + neutralizing anti-IL-4 antibody and ifosfamide (*F*). Bladders from the saline-treated group showed normal urothelium with no sign of edema (ed) or urothelial denudation (ud). Ifosfamide-injected bladders exhibited segmental urothelial denudation, severe edema with effacement of the submucosa and occasional frank hemorrhage. Ifosfamide-injured bladders exposed to H-IPSE, IL-4, or MESNA showed intact urothelium and overall preserved bladder architecture with a relative lack of edema. The protective effect of H-IPSE was reversed by prior administration of neutralizing anti-IL-4 antibody.

The bladders of mice treated with saline, H-IPSE, H-IPSE followed by neutralizing anti-IL-4 antibody, recombinant IL-4, or MESNA were subjected to histologic analyses, 12 h after ifosfamide injection. Compared with the bladder sections from mice given only saline and no ifosfamide (**Fig. 3*A***), we observed histologic evidence of urothelial denudation, lamina propria edema, and hemorrhage in the group of mice treated with only saline before ifosfamide (Fig. 3*B*). Conversely, groups of mice treated with MESNA (Fig. 3*C*) or IL-4 (Fig. 3*D*), as well as the H-IPSE-treated group (Fig. 3*E*), all showed significantly reduced bladder pathology, including more intact urothelium and decreased edema and hemorrhage. However, it was apparent that anti-IL-4 antibody reversed the protective effect of H-IPSE (Fig. 3*F*), as significant bladder pathology was observed in mice given both H-IPSE and anti-IL-4 antibody, akin to the ifosfamide-only group.

### H-IPSE attenuates spontaneous and evoked pain responses in ifosfamide-injected mice

**Figure 4:**
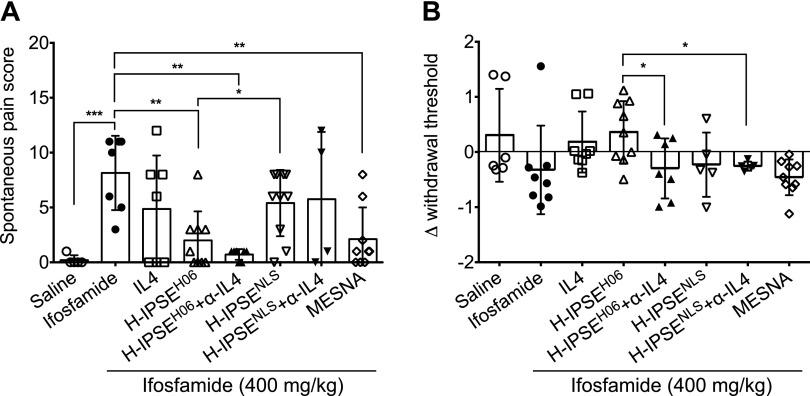
H-IPSE reduces spontaneous pain but not evoked tactile sensitivity in ifosfamide-treated mice. Mice were administered recombinant IL-4, H06 H-IPSE, H06 H-IPSE with neutralizing anti-IL-4 antibody (α-IL-4), MESNA, or saline before ifosfamide challenge. *A*) Animals were then placed individually in fresh cages with new bedding. Mice were evaluated and spontaneous pain scored (in a blinded fashion). Higher scores indicate more pain. *B*) Mice were subjected to tactile allodynia assessment using von Frey monofilaments. The change in withdrawal threshold is the difference between the 50% withdrawal threshold at baseline and 10-h postifosfamide injection. As a higher 50% threshold indicates less pain, positive values for changes in withdrawal threshold indicate less pain. The reduced pain observed in the H-IPSE- and IL-4-treated groups was not significant compared with the mice treated with only ifosfamide. The change in 50% threshold was significantly lower in the H-IPSE and H-IPSE NLS mutant groups when coadministered neutralizing anti-IL-4 antibody. The plotted data are pooled from 3 repeat experiments. Horizontal bars represent means. *P < 0.05, **P < 0.01, ***P < 0.001.

To determine whether H-IPSE also ameliorates CHC-associated pain, mice were evaluated for spontaneous pain characteristics, as well as von Frey monofilament-based evaluation of evoked pain. Whereas spontaneous pain was induced in all mice receiving ifosfamide, spontaneous pain scores were significantly reduced in the groups of mice injected with H-IPSE (*P* = 0.0007) and MESNA (*P* = 0.0016) compared with the mice receiving only ifosfamide (**Fig. 4*A***). Interestingly, the therapeutic effect of H-IPSE was absent or reduced in the group of mice given the NLS mutant of H-IPSE (*P* = 0.0161). Administration of neutralizing anti-IL-4 antibody did not reverse this protective effect of H-IPSE. Evaluations of allodynia (evoked pain responses) demonstrated that changes in withdrawal threshold observed for the H-IPSE and IL-4 groups were like the negative-control (saline-only, no ifosfamide) group but not statistically different from the ifosfamide-only and the H-IPSE NLS-mutant groups. However, the effect of H-IPSE on evoked pain was significantly reversed when neutralizing anti-IL-4 antibody was administered in addition to H-IPSE or its NLS-mutant (Fig. 4*B*).

### Effect of H-IPSE on voiding patterns in ifosfamide-exposed mice

**Figure 5:**
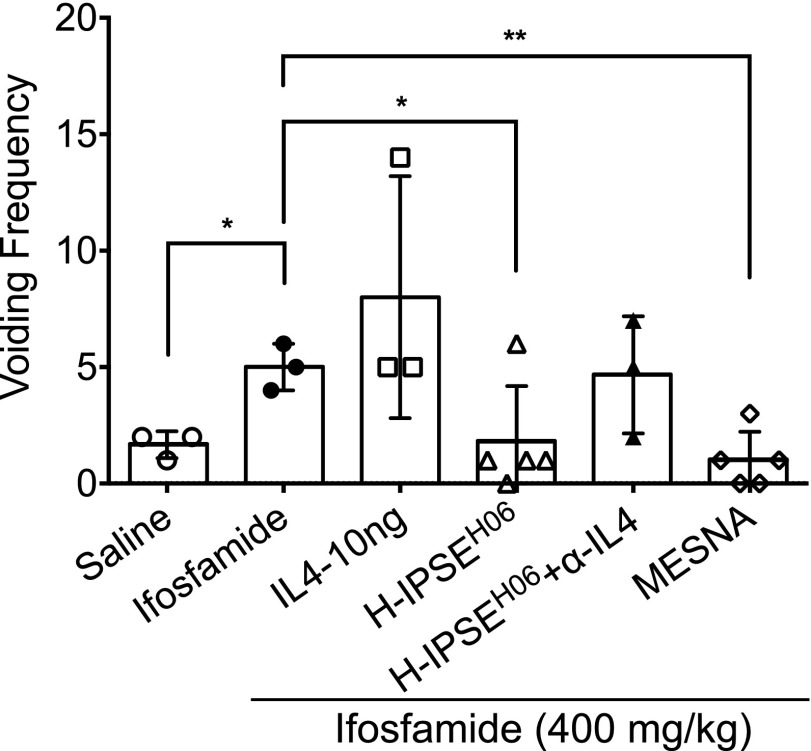
H-IPSE reduces abnormal voiding behaviors in ifosfamide-treated mice. Mice were subjected to the voided spot on paper test to measure their voiding frequency over a 4 h period. As compared with the mice exposed only to ifosfamide, mice administered H-IPSE and ifosfamide showed a significant reduction in voiding frequency. Administration of anti-IL-4 mAb seems to reverse the effect of H-IPSE. Plotted data are from 1 of 2 representative experiments. Horizontal bars represent means. *P < 0.05, **P < 0.01.

To determine the effect of H-IPSE injection on ifosfamide-induced abnormal voiding behaviors, changes in voiding frequency and voiding pattern were assessed through application of the voided spot assay on ifosfamide-exposed mice. Generally, the voiding patterns in the H-IPSE and MESNA-treated groups resembled the saline only negative control-treated group (**Fig. 5**, Supplemental Figs. 3 and 4). Voiding frequency was significantly increased in the ifosfamide-injected group relative to the saline only negative control group (*P* = 0.0132), H-IPSE-treated (*P* = 0.0406), and MESNA-treated (*P* = 0.0037) groups, respectively (Fig. 5). While the voiding frequency of mice administered neutralizing anti-IL-4 antibody after H-IPSE resembled that of mice given ifosfamide alone, administration of recombinant IL-4 to ifosfamide-exposed mice did not affect voiding frequency.

### H-IPSE restores normal gene expression of urothelial differentiation markers in ifosfamide-injured bladders in an IL-4- and NLS-dependent manner

**Figure 6:**
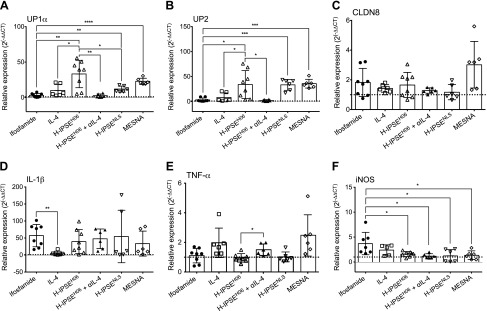
H-IPSE restores expression of uroplakin mRNA and reduces proinflammatory mediator mRNA expression in the ifosfamide-injured bladder. Total RNA was purified from the bladders of mice administered ifosfamide alone or ifosfamide in addition to the following: saline, recombinant IL-4, H-IPSE, the NLS mutant of H-IPSE, H-IPSE with neutralizing anti-IL-4 antibody (αIL-4), or MESNA. Real-time PCR was then performed to measure transcription of uroplakin genes [*Up1a* (*A*) and *Up2* (*B*)] and a tight junction-associated gene [*Cldn8* (*C*)]. After ifosfamide insult, H-IPSE-injected mice showed significantly increased transcript levels of the uroplakin genes *vs.* mice exposed to ifosfamide alone. Transcription of proinflammatory mediator genes [*Il-1β* (*D*), *Tnf-α* (*E*), and *iNOS* (*F*)] was also assessed. H-IPSE- and MESNA-injected mice showed significantly decreased transcript levels of iNOS compared with mice exposed only to ifosfamide. Ifosfamide-exposed mice, given the NLS mutant of H-IPSE or H-IPSE in combination with the anti-IL-4 antibody, still had reduced levels of *iNOS* gene expression compared with mice given ifosfamide alone. H-IPSE-injected mice also exhibited significantly reduced levels of *Tnf-α* *vs.* the group treated with the anti-IL-4 antibody but not *Il-1β* relative to mice exposed to ifosfamide alone. *P < 0.05, **P < 0.01, ***P < 0.001, ****P < 0.0001.

Uroplakins are urothelial-specific transmembrane proteins that function to maintain urinary-tract integrity and are markers of urothelial differentiation. It has been proposed that uroplakins may be the primary molecular target of ifosfamide/cyclophosphamide-induced acrolein and reactive oxygen species in the bladder (54). We measured levels of uroplakin as readout for urothelial denudation and surrogate for urothelial regeneration. Indeed, we observed significant decreases in bladder expression of the uroplakin genes *Up1α* (*P* = 0.0030) and *Up2* (*P* = 0.0174) in mice injected with ifosfamide, 12 h earlier, compared with mice given 1 intravenous dose of H-IPSE before ifosfamide (**Fig. 6*A, B***). In line with a previous report that MESNA preserves urothelial integrity and uroplakin expression (55), we also found significantly higher expression of *Up1α* (*P* < 0.0001) and *Up2* (*P* = 0.0001) in mice injected with 3 doses of MESNA at 0, 4, and 8 h postifosfamide exposure compared with the mice given saline before ifosfamide. Administration of the anti-IL-4 antibody to H-IPSE-injected mice before ifosfamide insult significantly reversed the protective effect of H-IPSE on *Up1α* (*P* = 0.0027) and *Up2* (*P* = 0.0143) expression compared with the H-IPSE-treated group. Although the NLS mutant-treated group featured lower expression of *Up1α* compared with the wild-type H-IPSE-treated group (*P* = 0.0167), the NLS mutant of H-IPSE induced higher expression of both *Up1α* (*P* = 0.0022) and *Up2* (*P* = 0.0007) compared with the mice given saline before ifosfamide (Fig. 6*A, B*). As previously reported, the natural urothelial-regenerative response after bladder insult sets in 12 h postchallenge (56, 57). This may explain why the expression of uroplakins was almost restored to baseline in the ifosfamide-only group, but the rate of such increase was significantly higher in the H-IPSE, MESNA, and IL-4 groups. In line with previous observations by Golubeva *et al.* (58), gene expression of the tight junction protein *Cldn8* (Fig. 6*C*) was neither significantly affected by H-IPSE injection nor ifosfamide exposure.

### H-IPSE affects gene expression of iNOS, but not IL-1β and TNF-α, in ifosfamide-exposed bladders in an IL-4- and NLS-independent fashion

The pathogenesis of CHC is mediated by the activities of soluble mediators, including proinflammatory cytokines, such as *Il-1β* and *Tnf-α*, NO production through the action of *iNOS*, prostaglandin production by *Cox-2* (2, 11–16), and chemokines. We assessed the effect of H-IPSE on the expression of representative proinflammatory mediators in ifosfamide-injected mice. All treatment groups receiving ifosfamide showed higher levels of *Il-1β* compared with the baseline levels seen in saline-injected, ifosfamide-naïve mice (dotted line in Fig. 6*D*). However, the recombinant IL-4-injected mice exhibited significantly reduced levels of *Il-1β* compared with the ifosfamide-only group. *Tnf-α* expression levels in H-IPSE were not significantly reduced relative to the ifosfamide group but were significantly lower in H-IPSE groups compared with the group given neutralizing anti-IL-4 antibody (Fig. 6*E*). Interestingly, the expression level of *iNOS* was significantly reduced in ifosfamide-exposed mice injected with H-IPSE, its NLS mutant, or MESNA, whether or not neutralizing anti-IL-4 antibody injection was coadministered (Fig. 6*F*). Furthermore, whereas the transcription levels of *Cox-2* and *Il-8* were not significantly changed, the level of gene expression of the macrophage inflammatory protein *Ccl3* was decreased in the H-IPSE groups regardless of NLS mutation or anti-IL-4 antibody treatment (Supplemental Fig. 5).

## DISCUSSION

Hemorrhagic cystitis is a form of severe bladder inflammation, which may result from various infectious and noninfectious causes (59). Regardless of underlying etiology, hemorrhagic cystitis can be associated with significant bladder hypersensitivity, including symptoms, such as urinary frequency, urgency, and bladder pain. Urogenital schistosomiasis is a classic infectious cause of hemorrhagic cystitis, whereas CHC is a major noninfectious trigger. Hematuria is a cardinal feature of schistosomiasis-associated hemorrhagic cystitis. It results from tissue damage as a result of granulomatous infiltration of leukocytes in response to antigens continuously released from eggs lodged in the bladder wall (60). However, only a minority of infected individuals shows day-to-day hematuria and urinary symptoms (30). Whereas several other factors, including intensity of infection, are associated with the degree of hematuria, it is possible that *S. hematobium*, the helminth that causes urogenital schistosomiasis, may be producing host modulatory factors to modulate host pathogenesis (61). Indeed, the interaction between secreted and parasite tegument-bound molecules and the host-immune response shapes the evolution of schistosome-associated immunopathogenesis (62). For urogenital schistosomiasis, parasite factors released from the trapped egg may mediate the balance between egg passage through the tissue to complete the life cycle and cause life-threatening pathogenesis (which is not in the parasite’s “interests”). IPSE is the most abundant secreted protein from *S. mansoni* eggs (60). M-IPSE has been shown to bind Igs, ligate IgE on the surface of basophils and mast cells to induce these cells to release IL-4 (32–34), and sequester chemokines (35) and is an infiltrin that can translocate into host nuclei to modulate host gene transcription (36–38). Until now, it was unclear whether these molecular functions of IPSE could be therapeutically exploited in inflammation-mediated, clinically relevant diseases. Here, we report the first therapeutic application of a uropathogen-derived molecule in models of bladder-specific pathology, namely chemotherapy-induced bladder hypersensitivity and hemorrhagic cystitis.

Ifosfamide and cyclophosphamide are nitrogen mustard alkylating agents used for treatment of many cancers (1, 63, 64). However, these agents are associated with a range of life-threatening side-effects, including hemorrhagic cystitis, arrhythmias, neuro- and nephrotoxicity, Fanconi syndrome, and even urothelial cell carcinoma (7, 64, 65). MESNA is used to prevent CHC but fails to treat hemorrhagic cystitis after its onset (5, 66, 67). Other options for managing this debilitating chemotherapy-induced condition, including use of recombinant IL-4 (10), which has been shown to have a therapeutic effect in experimental models, and surgical solutions are often suboptimal and sometimes lead to other untoward, severe, adverse reactions (17–28). Thus, there is need for novel therapeutic options for hemorrhagic cystitis.

Inspiration to exploit urogenital parasite-derived, host-modulatory molecules as potential therapeutics has been provided by recent advances in the use of gastrointestinal parasite modulatory proteins as therapy for allergic diseases and autoimmune disorders. There is ongoing research involving administration of crude *Trichuris trichiura* or hookworm ova or worm extracts to treat inflammatory bowel diseases, such as ulcerative colitis and Crohn’s disease, and autoimmune disorders, such as multiple sclerosis and celiac disease (68–73). In this report, we show that a uropathogenic parasite-derived molecule (H-IPSE) can ameliorate hemorrhagic cystitis, specifically by reducing bladder hemorrhage, urothelial denudation, ulceration, and associated pain.

A single intravenous injection of H-IPSE was more effective than multiple doses of MESNA, the current standard of care for preventing CHC, in reducing ifosfamide-induced bladder hemorrhage, as measured by bladder hemoglobin content. Based on the described functions of M-IPSE, the immediate target of intravenously administered H-IPSE may be circulating basophils. M-IPSE binds IgE on the surface of these cells to induce IL-4 release (32–34). IL-4 induces an anti-inflammatory phenotype *via* inhibition of the production and release of proinflammatory cytokines and mediators, such as TNF-α, IL-1β, iNOS, and COX-2, and thereby alleviates inflammation-driven diseases (2, 10–16). Indeed, our data showed that the ability of H-IPSE to reduce ifosfamide-triggered bladder hemorrhage relies on an IL-4-dependent mechanism. The reversal of the effect of H-IPSE by the NLS mutant was not statistically significant.

Bladder expression of iNOS was reduced in groups of ifosfamide-exposed mice receiving the NLS mutant or H-IPSE, either with or without neutralizing anti-IL-4 antibody. It is possible that infiltrating cells and urothelial cells are a source of iNOS that can be altered by H-IPSE exposure. The capacity of H-IPSE to sequester proinflammatory chemokines (35) may prevent recruitment and infiltration of inflammatory cells (74–76). We speculate that the observed ameliorative effect, although independent of IL-4- and NLS-linked mechanisms, may involve other mechanisms (such as those mediated by chemokines) in multipronged or synergistic interactions.

H-IPSE increased transcription of uroplakin genes in the ifosfamide-exposed bladders in an IL-4- and NLS-dependent manner. As expected in this setting, MESNA also maintained the gene-expression level of uroplakins (55). Uroplakin gene-expression levels in ifosfamide-injured bladders were significantly higher in the H-IPSE- *vs.* IL-4-treated group. This suggests that H-IPSE induces higher IL-4 levels than that achieved by administration of recombinant IL-4 and/or that H-IPSE may have direct or indirect IL-4-independent effects on urothelial cells that promote repair responses. The uroplakins are not only markers of urothelial differentiation but also serve as barrier function proteins that protect the urothelium and deeper bladder tissues from damage (57). Not surprisingly, gene expression of all of the uroplakin genes (*Up1α*, *Up1β*, *Up2*, *Up3α*, *Up3β*) reaches a nadir after chemical or biologic insult (57). Specifically, transcription of these genes achieves maximal reductions within the first 12 h after ifosfamide/cyclophosphamide challenge, before the onset of urothelial regeneration (56, 57). Given that the bladder-expression level of uroplakin genes in the H-IPSE group was comparable or higher than the levels seen in the MESNA-treated group, it is possible that H-IPSE triggers the same or similar mechanisms of urothelial repair as MESNA. Indeed, we have found that H-IPSE induces urothelial cell proliferation *in vitro* in an NLS-dependent manner in another study. These findings are consistent with a role for H-IPSE in promoting urothelial regeneration through IL-4-independent, NLS-dependent pathways.

Finally, although the observed ameliorative effect of H-IPSE on hemorrhagic cystitis and associated pathogenesis is compelling, the exact molecular mechanisms underlying the protective effect are still not fully delineated. For instance, we do not know why H-IPSE decreases ifosfamide-induced bladder hemorrhage but not edema. It is also unknown why H-IPSE significantly decreases spontaneous but not evoked pain in ifosfamide-exposed mice. Finally, it is unclear how H-IPSE mediates a profound therapeutic effect in the ifosfamide-injured bladders despite not significantly affecting TNF-α or IL-1β gene expression. However, the fact that iNOS expression was decreased in all H-IPSE groups may suggest that the timing of the assay may have missed upstream events in the proinflammatory pathway, in addition to the early onset of urothelial repair following chemical insult. Ongoing work is focusing on more comprehensive transcriptional profiling of the IPSE-treated, ifosfamide-injured bladders, in addition to generating other H-IPSE mutants that will lead to enhanced efficacy while minimizing potential toxicity. Development of H-IPSE as a safe therapeutic will need to address the possibility of histamine release by IgE^+^ basophil and mast cell degranulation induced by H-IPSE binding of IgE.

Other potential pitfalls exist in our experimental approaches. Although we used a well-established clone for anti-IL-4 antibody-mediated neutralization of this cytokine, this tool does not allow us to link directly H-IPSE-induced activation of IgE-bearing basophils/mast cells to IL-4 production. We also currently lack a means by which to neutralize the chemokine-binding properties of H-IPSE. Ongoing efforts are directed toward addressing these shortcomings. Finally, although NLS is an important mediator of IPSE translocation into host cell nuclei, the NLS mutant of H-IPSE is still capable of reaching nuclei, albeit at a much lower efficiency than the wild-type protein (38). Thus, our observed effects of the NLS mutant may have been dampened by this residual form of nuclear transport.

In summary, H-IPSE, a uropathogen-derived molecule, alleviates CHC in a mouse model in an IL-4-dependent fashion. H-IPSE may prove more efficacious than MESNA, the current standard of care for prophylaxis against this devastating complication of chemotherapy. Our H-IPSE mammalian expression system may poise us for large-scale protein production that would be necessary for expanded testing of H-IPSE as a therapeutic molecule.

## Supplementary Material

This article includes supplemental data. Please visit *http://www.fasebj.org* to obtain this information.

## Abbreviations

4get: IL-4/green fluorescent protein-enhanced transcript
CHC: chemotherapy-induced hemorrhagic cystitis
Cldn8: claudin 8
Cox-2: cyclooxygenase 2
GFP: green fluorescent protein
H-IPSE: *Schistosoma hematobium* homolog of IL-4-inducing principle from *Schistosoma mansoni* eggs
IHC: ifosfamide-induced hemorrhagic cystitis
IPSE: IL-4-inducing principle from *Schistosoma mansoni* eggs
M-IPSE: *Schistosoma mansoni* homolog of IL-4-inducing principle from *Schistosoma mansoni* eggs
MESNA: 2-mercaptoethane sulfonate sodium
NLS: nuclear localization sequence
RBC: red blood cell
Up1a/2: uroplakin 1a/2
