# Supplementary material for: Therapeutic exploitation of IPSE, a urogenital parasite-derived host modulatory protein, for chemotherapy-induced hemorrhagic cystitis
Source: FASEB J. 2018 Apr 3;32(8):4408–19. doi: 10.1096/fj.201701415R (PMC6044057; doi:10.1096/fj.201701415R)
Supplement: Supplementary file 1 [file fj.201701415R.sd1.pdf]

Supplementary data

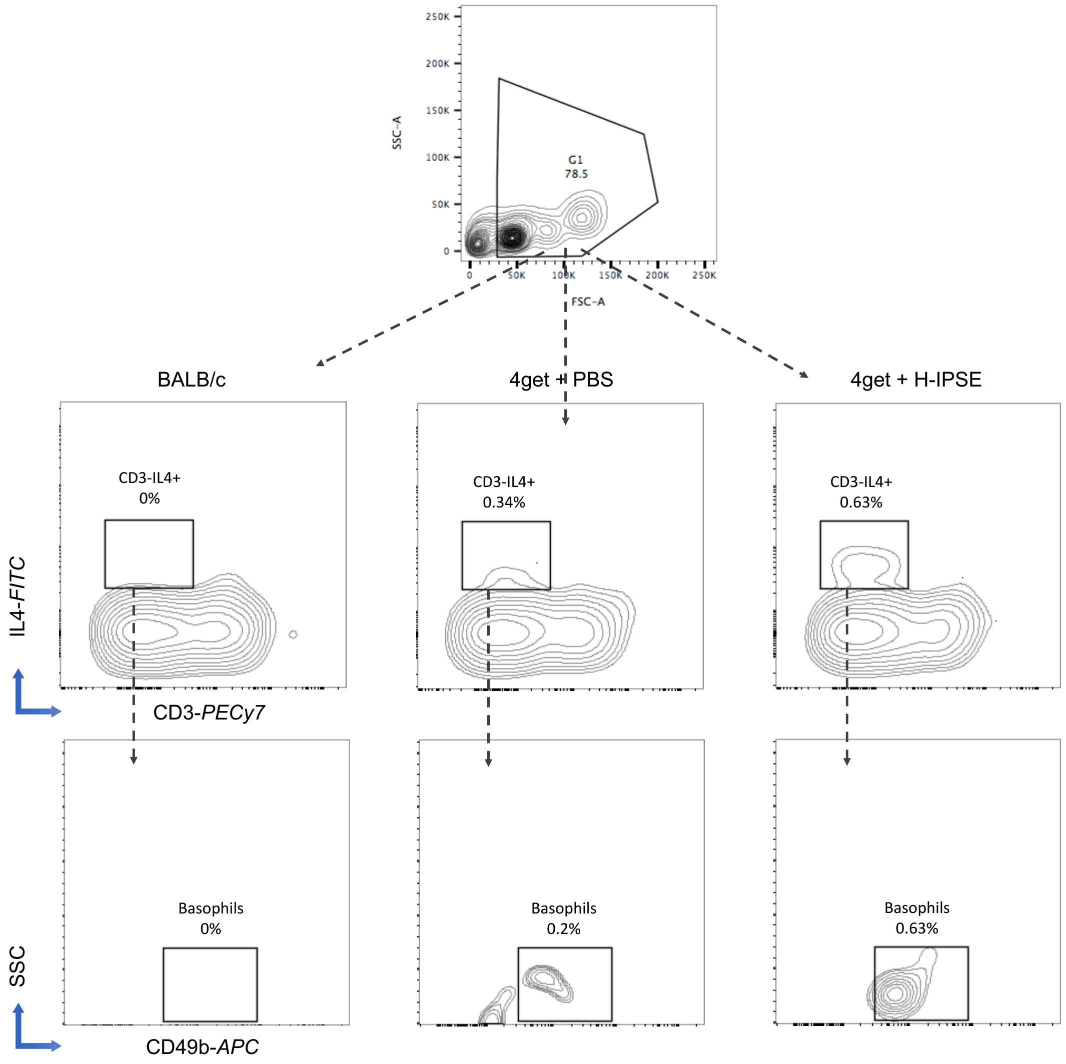

Figure S1. Flow cytometry gating *strategy for 4get mice.* Representative flow cytometric gating strategy and contour plots demonstrating that H-IPSE stimulates IL-4 transcription by basophils. 4get mice were intravenously injected with recombinant H-IPSE, 24 hours prior to flow cytometric analysis to assess the proportion of CD3-CD49b+ cells (basophils) and CD3-c-kit+ cells (mast cells) that express IL-4.

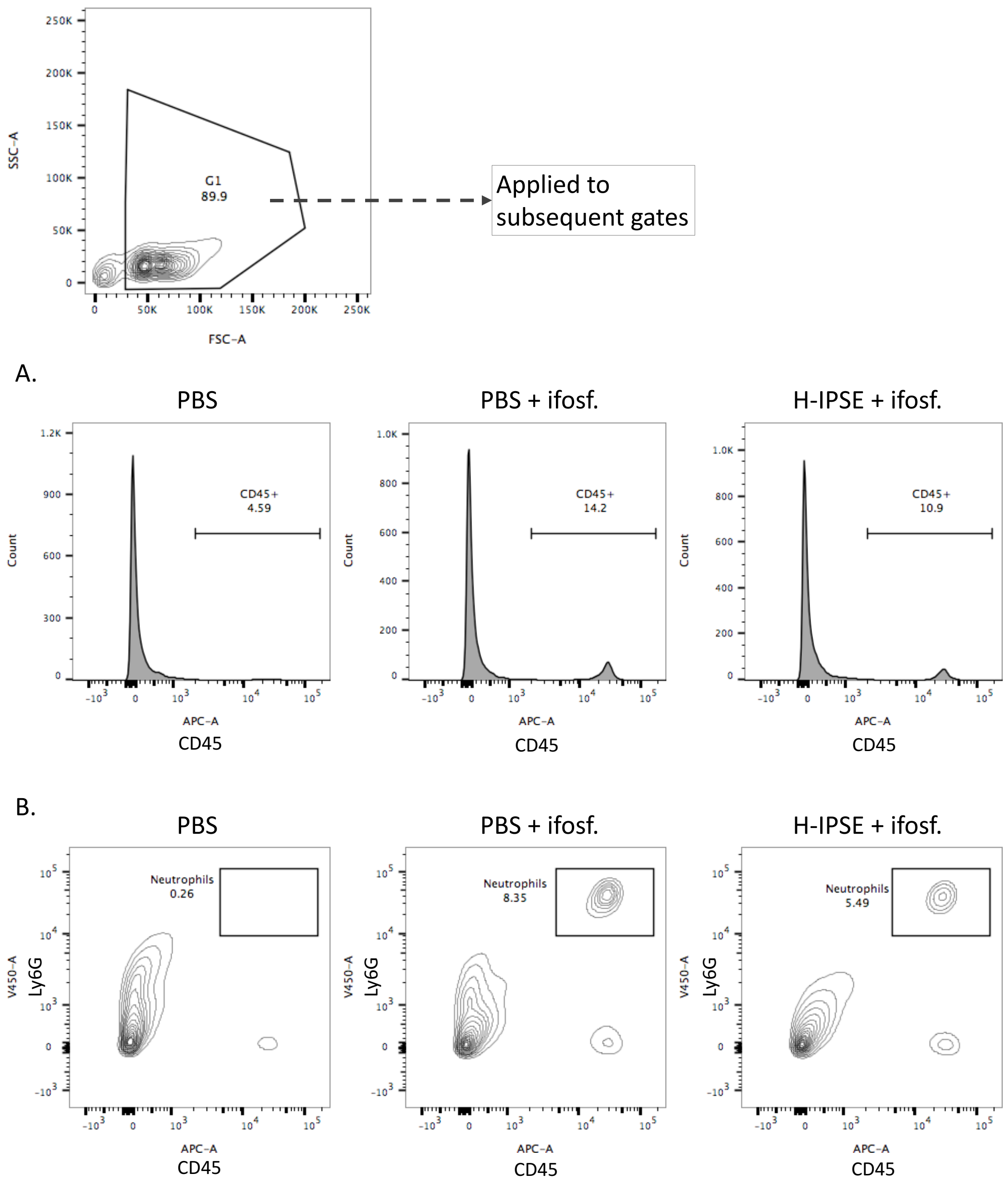

Figure S2. Treatment with H06 H-IPSE before ifosfamide injection reduced bladder leukocyte infiltration. *Mice were intravenously given either H06 H-IPSE or PBS 24 hours before ifosfamide injection. There was an approximately 50% decrease in the proportion of bladder infiltrating leucocytes and neutrophils in the H-IPSE treated group as compared to the PBS positive control group.*

H-IPSE Reduces Abnormal Voiding Behaviors Due to Ifosfamide-Induced HC

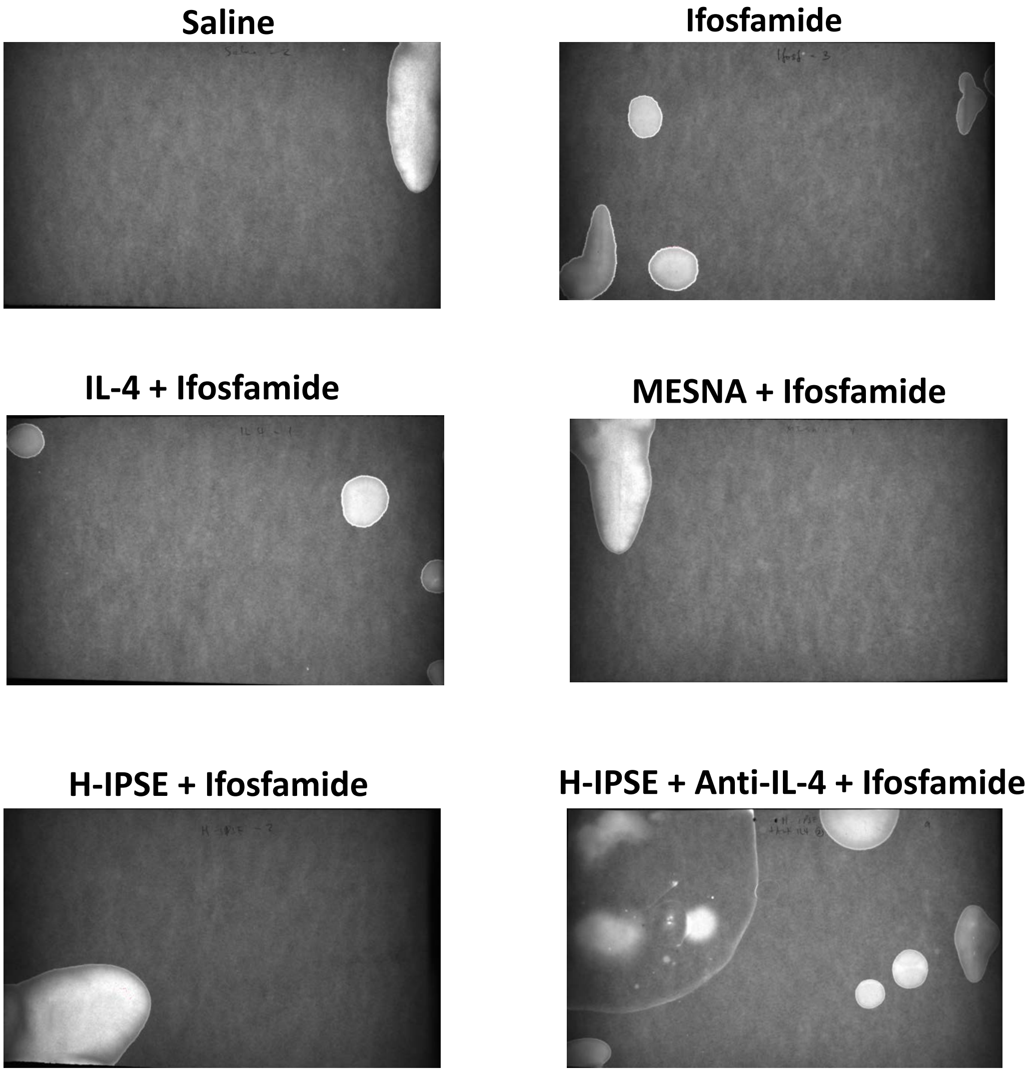

Figure S3. Effect of H-IPSE on abnormal voiding behavior in ifosfamide injected animals. Photographs of representative UV trans-illuminated filter papers showing decreased voiding frequency and reduced abnormal voiding patterns in H-IPSE, IL-4, and MESNA injected mice as compared to the mice exposed to ifosfamide only and to mice administered anti-IL-4 antibody after H-IPSE injection and ifosfamide exposure.

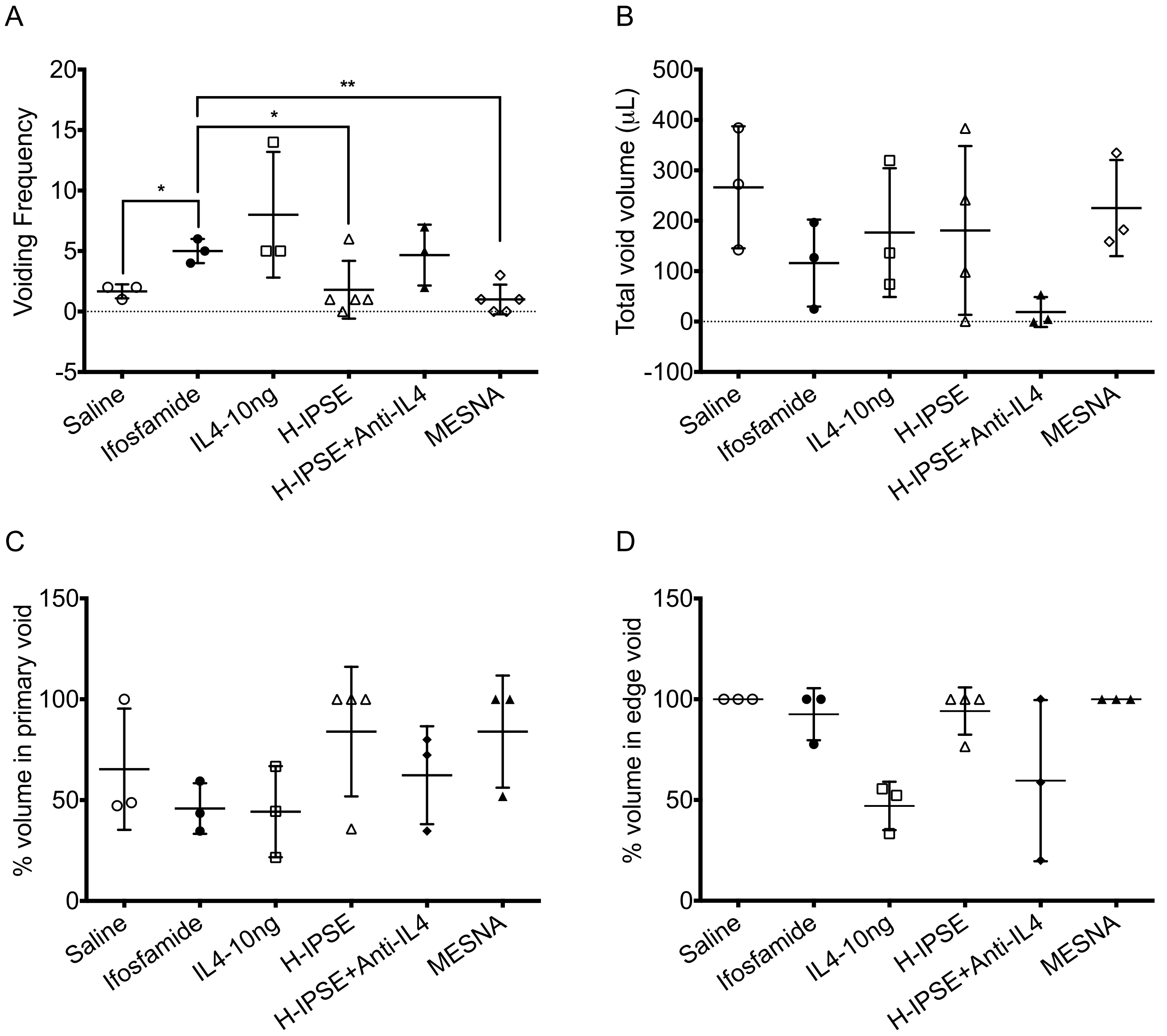

Figure S4. H-IPSE reduces abnormal voiding behaviors in ifosfamide injected animals in an IL-4 dependent manner. Mice injected with H-IPSE or MESNA before ifosfamide showed reduced voiding frequency and normal voiding patterns (void volume, volume in primary void and edge void), akin to the negative control, and as compared to the ifosfamide only group.

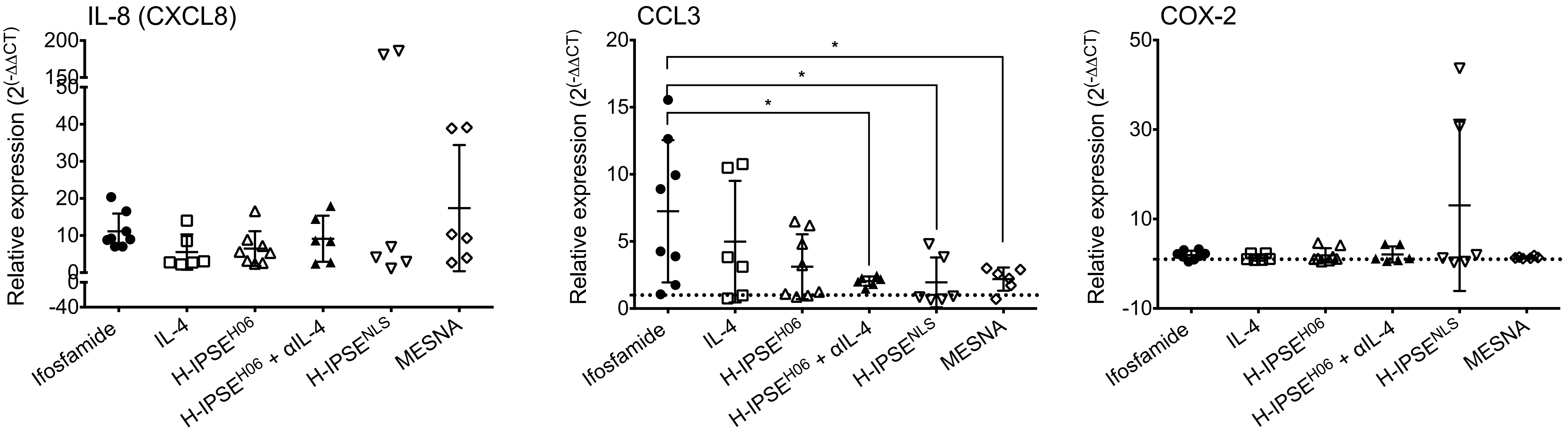

Figure S5. Expression of other pro-inflammatory mediators in ifosfamide-injected mice. IL-8 and Cox-2 were not significantly altered but CCL3 was reduced in all H-IPSE injected groups with or without the NLS-mutation and neutralizing anti-IL-4 treatment, albeit not significant for H-IPSE alone.

Table S1. List of Primers used for real time PCR

**Gene name**	Forward Primer sequence	Reverse Primer sequence
Upk1a	5’-TACACCCACCGCGACTATATG-3’	5’-CCACAACACTCTTGCTCAATCAT-3’
Upk2	5’-TGCCCCTGATCCTGATTCTG-3’	5’-CAAGGCAATTAACAGGCTTTCTG-3’
CLDN8	5’-AGAGCCGCATCTTGCTGAC-3’	5’-TCTGATGATGGAATTGGCAACC-3’
IL-1b	5’-GAAATGCCACCTTTTGACAGTG-3’	5’-TGGATGCTCTCATCAGGACAG-3’
TNF-a	5’-GTGGAACTGGCAGAAGAG-3’	5’-CCATAGAACTGATGAGAGG-3’
iNOS	5’-ACATCGACCCGTCCACAGTAT-3’	5’-CAGAGGGGTAGGCTTGTCTC-3’
IL-8	5’-CACCTCAAGAACATCCAGAGCT-3’	5’-CAAGCAGAACTGAACTACCATCG-3’
CCL3	5’-CAGCCAGGTGTCATTTTCCT-3’	5’-CTGGCTCCAAGACTCTCAGG -3’
COX-2	5’-CAAGGGAGTCTGGAACATTG-3’	5’-ACCCAGGTCCTCGCTTATGA-3’
GAPDH	5’-AGGTCGGTGTGAACGGATTTG-3’	5’-TGTAGACCATGTAGTTGAGGTCA-3’
